# Total extraperitoneal endoscopic hernioplasty (TEP) versus Lichtenstein hernioplasty: a systematic review by updated traditional and cumulative meta-analysis of randomised-controlled trials

**DOI:** 10.1007/s10029-019-02049-w

**Published:** 2019-10-10

**Authors:** P. Gavriilidis, R. J. Davies, J. Wheeler, N. de’Angelis, S. Di Saverio

**Affiliations:** 1 Division of Gastrointestinal and Hepato-Biliary-Pancreatic Surgery, Imperial College Healthcare NHS Trust, Hammersmith Hospital, London, W12 0HS UK; 2 Cambridge Colorectal Unit, Addenbrooke’s Hospital, Cambridge University Hospitals NHS Foundation Trust, Hills Road, Cambridge, CB2 0QQ UK; 3 Department of Digestive Surgery, Henri Mondor University Hospital, 94010 Créteil, France

**Keywords:** Total extraperitoneal hernioplasty, TEPP, Lichtenstein technique, Inguinal hernia repair, Mesh, Hernia repair, Groin hernia

## Abstract

**Background–purpose:**

Totally extraperitoneal (TEP) endoscopic hernioplasty and Lichtenstein hernioplasty are the most commonly used approaches for inguinal hernia repair. However, current evidence on which is the preferred approach is inconclusive. This updated meta-analysis was conducted to track the accumulation of evidence over time.

**Methods:**

Studies were identified by a systematic literature search of the EMBASE, PubMed, Cochrane Library, and Google Scholar databases. Fixed- and random-effects models were used to cumulatively assess the accumulation of evidence over time.

**Results:**

The TEP cohort showed significantly higher rates of recurrences and vascular injuries compared to the Lichtenstein cohort; [Peto Odds ratio (OR) = 1.58 (1.22, 2.04), *p* = 0.005], [Peto OR = 2.49 (1.05, 5.88), *p* = 0.04], respectively. In contrast, haematoma formation rate, time to return to usual activities, and local paraesthesia were significantly lower in the TEP cohort compared to the Lichtenstein cohort; [Peto OR = 0.26 (0.16, 0.41), *p* ≤ 0.001], [mean difference = − 6.32 (− 8.17, − 4.48), *p* ≤ 0.001], [Peto OR = 0.26 (0.17, 0.40), *p* ≤ 0.001], respectively.

**Conclusions:**

This study, which is based on randomised-controlled trials (RCTs) of high quality, showed significantly higher rates of recurrences and vascular injuries in the TEP cohort than in the Lichtenstein cohort. In contrast, rate of postoperative haematoma formation, local paraesthesia, and time to return to usual activities were significantly lower in the TEP cohort than in the Lichtenstein cohort. Future multicentre RCTs with strict adherence to the standards recommended in the Consolidated Standards of Reporting Trials guidelines will shed further light on the topic.

**Electronic supplementary material:**

The online version of this article (10.1007/s10029-019-02049-w) contains supplementary material, which is available to authorized users.

## Introduction

Inguinal hernia repair is the most common operation in general surgery with more than 20 million performed annually worldwide [1]. Most patients with an inguinal hernia are symptomatic and the treatment of choice is surgical repair with mesh using open or laparo-endoscopic approach. The use of mesh varies worldwide from 0 to 5% in low-resource countries to 95% in high-resource countries. The Swedish National registry reported that for the year 2015, the percentages of inguinal hernia repair techniques were as follows: Lichtenstein hernioplasty 64%, totally extraperitoneal (TEP) hernioplasty 25%, transabdominal preperitoneal (TAPP) hernioplasty 3%, open preperitoneal hernioplasty 3.3%, and tissue repair 0.8%. The German Herniamed registry reported the following data for the period from 2009 to 2016: TAPP hernioplasty 39%, TEP hernioplasty 25%, and Lichtenstein hernioplasty 24%. There is a lack of data from America and Asia [1].

Some possible complications of hernioplasty include recurrence necessitating reoperations in 10–15% of cases and chronic pain (lasting more than 3 months) in 10–12% of cases, which may lead to long-term disability [1].

To date, the evidence comparing TEP hernioplasty to Lichtenstein hernioplasty is non-conclusive [2, 3]. However, there has been new published evidence since the most recent meta-analysis. Therefore, we decided to perform an updated traditional and cumulative meta-analysis to estimate the impact of the new studies on the robustness of the statistical significance of existing meta-analyses comparing TEP hernioplasty and Lichtenstein hernioplasty. Recurrence rate and chronic persistent pain were selected as primary outcomes.

## Methods

The preferred reporting items for systematic reviews and meta-analyses’ checklist was followed in this study [4].

### Literature search

With the use of the search terms in the free text and Medical Subject Headings terms (“laparoscopic or endoscopic total extraperitoneal inguinal repair”, “laparoscopic or endoscopic total extraperitoneal inguinal hernioplasty”, “open with mesh Lichtenstein inguinal hernia repair”, “Lichtenstein’s technique”, “inguinal hernia repair with mesh”, “TEP”, “inguinal hernia”, or “randomised or randomized controlled trial”), a systematic search of literature published over the last 30 years was performed using the EMBASE, Medline (PubMed), Cochrane Library, and Google Scholar databases. A grey literature search was also performed in the clinicaltrials.gov website. References of the retrieved articles were checked manually for additional studies. Disagreements between the authors were resolved by consensus-based discussions.

### Study selection, and inclusion and exclusion criteria

Only randomised-controlled trials (RCTs) that compared TEP laparoscopic inguinal hernia repair with Lichtenstein’s technique for inguinal hernia repair were included in this study. They fulfilled the following criteria: (1) clearly documented comparison of TEP laparoscopic approach and Lichtenstein’s technique for inguinal hernia repair, (2) report of at least one outcome measure, (3) inclusion of only the most recent publication in cases of multiple publications by the same institution, and (4) selection of TEP and Lichtenstein approaches from multi-arm RCTs.

Abstracts, retrospective studies, and non-English language publications were excluded from the analysis.

### Data extraction and outcomes

Two reviewers (PG and NA) independently extracted the following summary data from the included studies: name of authors; year of publication; number of patients included in the TEP and Lichtenstein hernioplasty cohorts; duration of operation; conversion rate; rates of haematoma and seroma formation; incidences of wound infection, vascular injury, and visceral injury; time to return to usual activities; incidence of persisting pain or persisting numbness; and recurrence rate.

### Definitions

Hernia recurrence was defined as any symptomatic or asymptomatic palpable lump or weakness in the operated groin found by the patient or the examining physician and exacerbated by the Valsalva manoeuvre. Chronic persisting pain was defined as pain of any severity (including testicular) persisting for more than 3 months after the operation. Impaired sensibility was defined as loss of the ability to register touch or the presence of numbness and tingling. Wound infection, vascular injury, and visceral injury were reported according to the definitions provided by the authors of the included studies. Operative time was defined as the time from the initial operative scalpel-to-skin contact to the placement of the last suture. Haematomas included wound and scrotal haematomas or ecchymoses but not bruising, and seromas included hydroceles. Time to return to usual activities was defined as the time taken to get back to normal social activities or work.

### Statistical analysis

The methodological quality of all included RCTs was based on Cochrane’s criteria, which include random sequence generation, allocation concealment, blinding of participants and personnel, blinding of outcome assessment, incomplete outcome data, selective reporting, and differences in baseline characteristics [5].

Statistical analysis was conducted using the STATA software (version 15, Stata Corp LP, College Station, TX, USA) and the Review Manager 5.3 software (Cochrane Collaboration, Oxford, England). Heterogeneity was assessed using the *I*^2^ test, and cut-off values of 25%, 50%, and 75% were considered of low, moderate, and high heterogeneity, respectively [6]. Where heterogeneity occurred, both fixed- and random-effects models were generated, and the conclusions compared, with the latter used where there were discrepancies. Fixed-effects models were used in cases of *I*^2^ value less than 25%.

Dichotomous variables were analysed based on odds ratios (ORs) with 95% confidence intervals. For the outcomes considered, the reference categories were selected, such that OR < 1 TEP.

Continuous variables were combined based on the mean difference (MD) and the standardised MD. The studies were then combined using the Mantel–Haenszel method in the first instance, with the Peto approach used when the cross-table has a zero cell [5, 6]. For studies that did not report the means and variances of the two groups, these values were estimated from the median, range, and the size of sample, using the technique described by Hozo et al. where possible [7].

In all analyses, the point estimate was considered significant at *P *< 0.05.

### Sensitivity analysis

Analyses of both primary and secondary outcomes were calculated using the random-effects and fixed-effect models to assess the impact of heterogeneity on the robustness of the conclusions. Cumulative analysis was performed to track the accumulation of evidence and to determine if the results of the meta-analysis were dominated by a particular study [8].

## Results

### Search strategy and included study characteristics

**Fig. 1:**
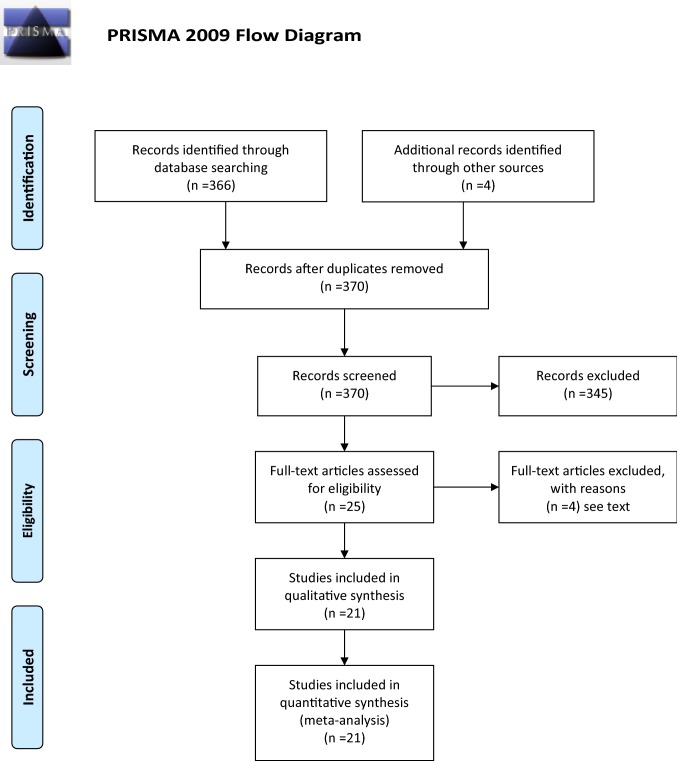
Diagram of search strategy

Twenty-one studies including 6573 patients were selected from a pool of 366 studies (Fig. 1). Of these patients, 3242 (49.3%) and 3331 (50.7%) underwent TEP and Lichtenstein hernioplasty, respectively [9–29]. Two abstracts, 1 in German and 1 in Spanish articles, were excluded. Non-significant differences were found in the demographic characteristics between the two cohorts (Table 1).

**Table 1:**
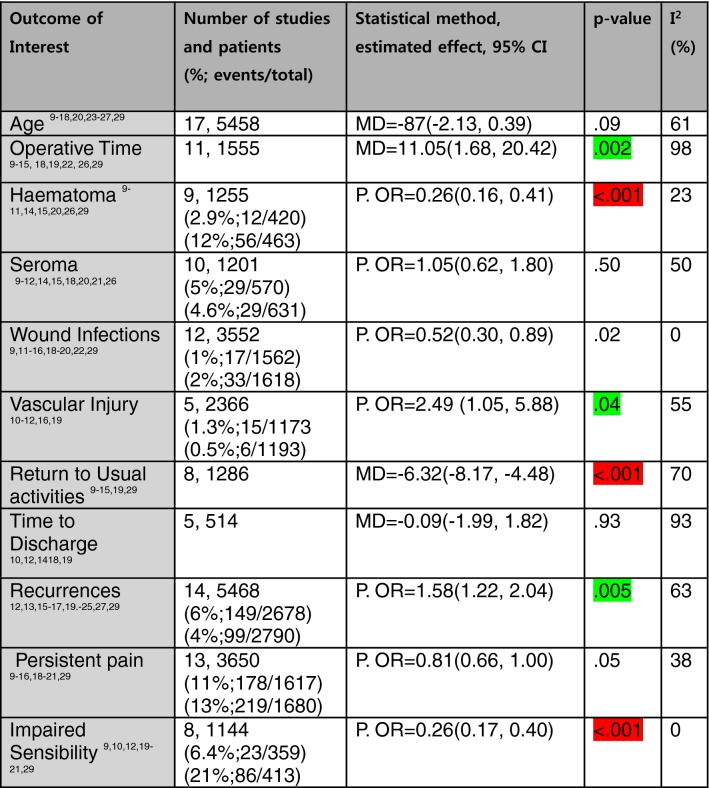
Outcomes of Interest

*P. OR* Peto odds ratio, *MD* mean difference, *CI* confidence intervals

Green highlighted favours Lichtenstein; red highlighted favours TEP; *I*^2^: heterogeneity metric

### Quality assessment of included RCTs

The methodological quality of the RCTs was poor; only 4 of the 21 studies blinded participants and personnel, and one of them blinded the assessors of the outcomes (Table 2).

**Table 2:** Risk of bias of RCTs

Author	Random sequence generation	Allocation concealment	Blinding of participants and personnel	Blinding of outcome assessment	Incomplete outcome data	Selective reporting
Wright	Low	Low	High	High	High	Unclear
Heikkinen et al. (1998) [10]	Low	Low	Unclear	High	Low	Low
Gokalp	Low	High	High	High	Low	Low
Andersson	Low	Low	High	High	Low	Low
Colak	Low	Low	High	High	Unclear	Low
Lal P	Unclear	Low	Unclear	Unclear	Unclear	High
Bringman	Low	Low	Unclear	High	Low	Low
Neumayer	Low	Unclear	Unclear	High	Low	Low
Heikkinen et al. (2004) [17]	Low	Low	Unclear	High	Low	Low
Lau	Unclear	High	Unclear	High	Low	High
Dedemadi	Low	Low	Low	Unclear	Low	Low
Pokorny	Low	Low	Low	Unclear	Low	Low
Hallén	High	High	High	Unclear	Low	Low
Eklund	Low	Low	High	High	Low	Low
Kuhia	Low	Low	Low	Unclear	Low	Low
Hamza	Low	High	High	High	Low	Low
Eker	Low	Low	Unclear	Unclear	Low	Low
Dhankhar	Low	Low	High	High	Low	Low
Wang	High	High	High	High	Unclear	Unclear
Moreno-Egea	Unclear	Low	Unclear	High	Low	Low
Gutlic	Low	Low	Low	Low	Low	Low
Pooled estimates	Low-risk 16 studies	Low-risk 15 studies	Low-risk 4 studies	Low-risk 1 study	Low-risk 17 studies	Low-risk 17 studies

### Primary outcomes

#### Recurrences

**Fig. 2:**
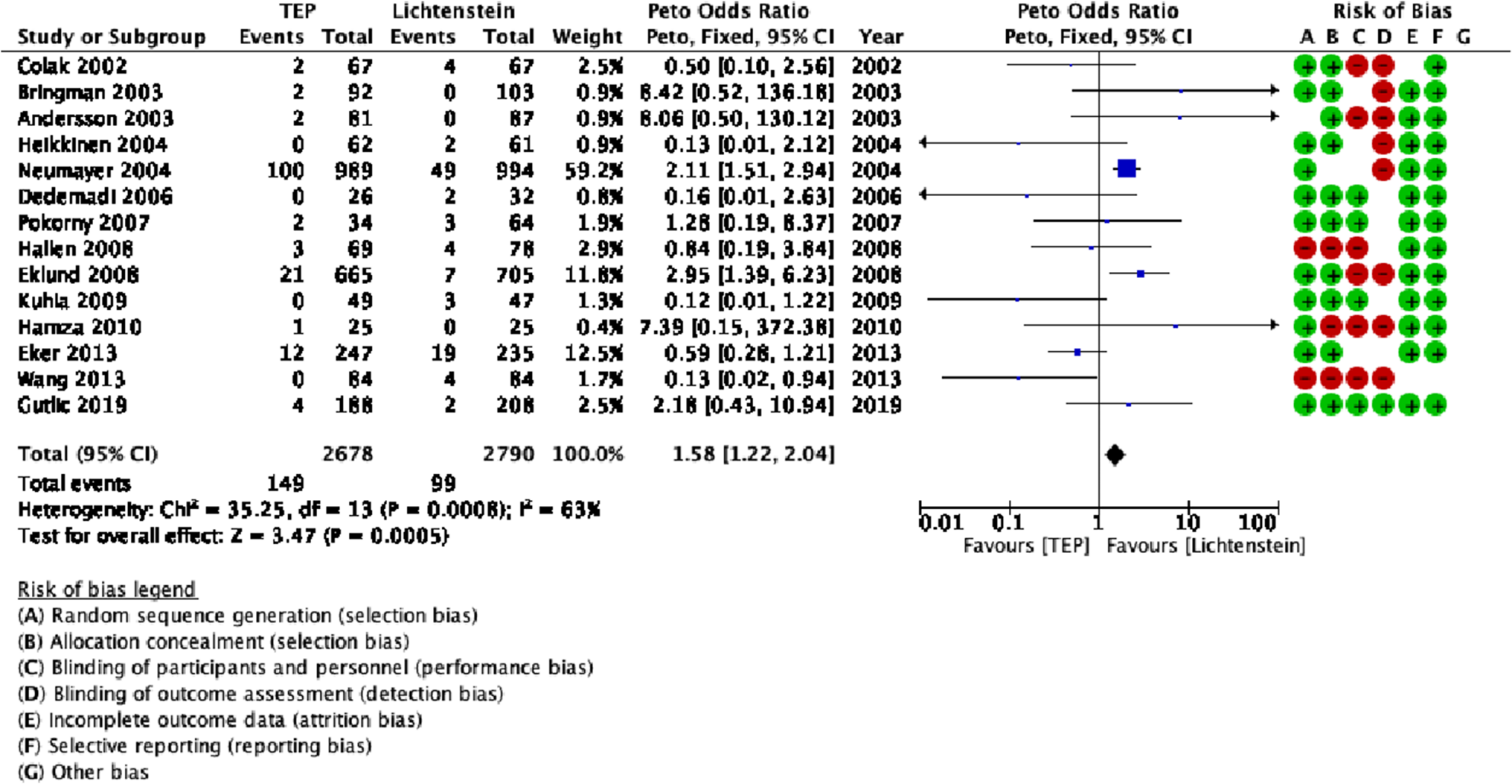
Forest plot of recurrences

There was evidence of a higher recurrences in the TEP cohort (149/2678 patients; 6% of patients) compared with the Lichtenstein cohort (99/2790 patients; 4% of patients) [Peto OR = 1.58 (1.22, 2.04), *p* = 0.005] (Table 1, Fig. 2).

#### Chronic persistent pain

There was no difference in chronic pain between the TEP cohort (185/1617 patients; 11% of patients) and the Lichtenstein cohort (228/1862 patients; 13% of patients) [Peto OR = 0.81 (0.66, 1.00), *p* = 0.05] (Table 1).

### Statistically significant secondary outcomes

There was evidence that the operative time was significantly shorter (by 11 min) in the Lichtenstein cohort than in the TEP cohort. Vascular injuries were significantly less in the Lichtenstein cohort than in the TEP cohort (Table 1).

There was evidence that the outcomes of haematoma formation rate, return to usual activities, and local paraesthesia were significantly better in the TEP cohort than in the Lichtenstein cohort (Table 1).

### Statistically non-significant secondary outcomes

Non-significant differences were observed in the outcomes of seroma formation rate, incidence of wound infections, and time to discharge (Table 1).

### Sensitivity analysis

**Fig. 3:**
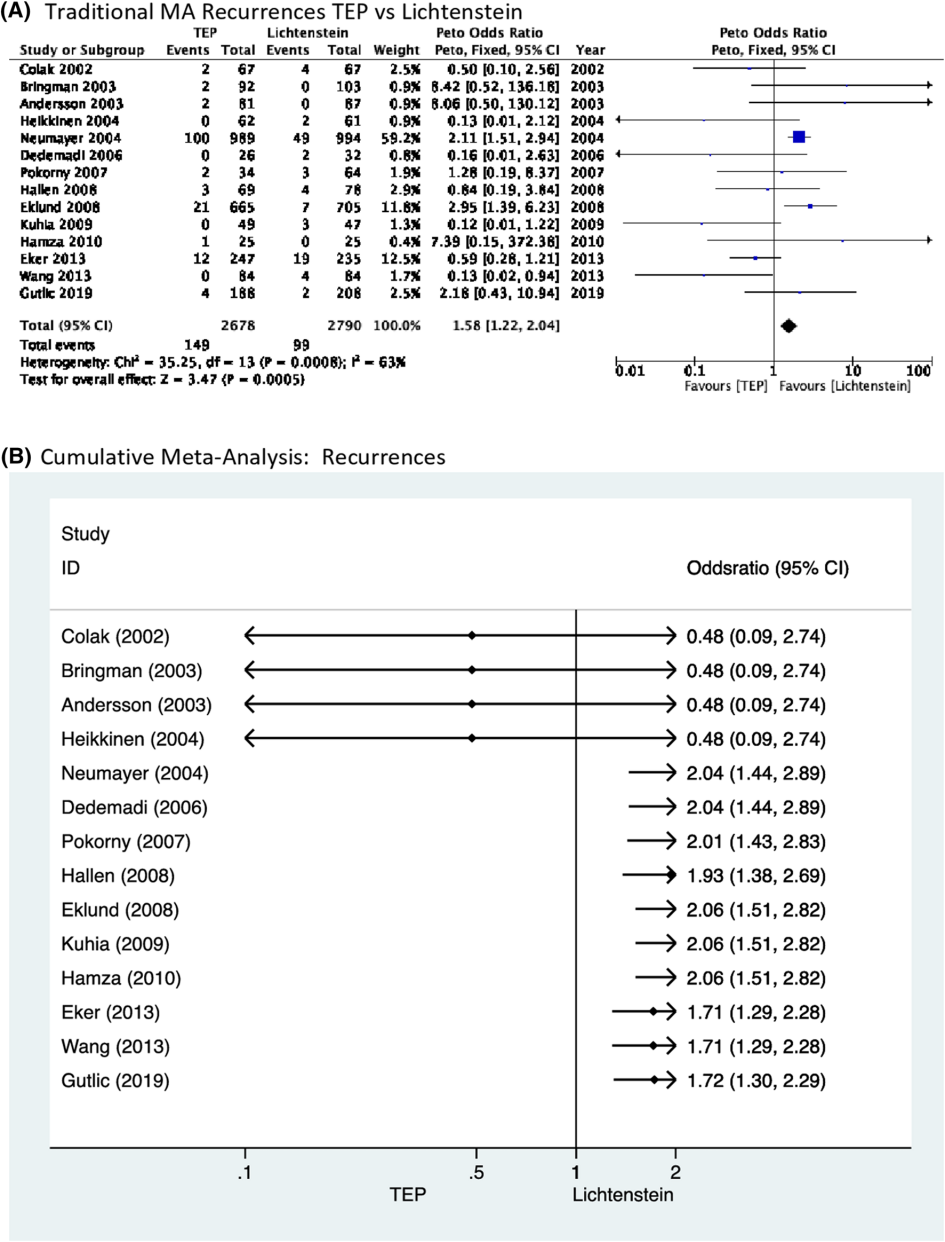
**a** Traditional meta-analysis of recurrences; **b** cumulative meta-analysis of recurrences

Analysis of outcomes using fixed- and random-effects models did not reveal any discrepancies. Cumulative meta-analysis further supports the evidence that the recurrence rate was significantly lower in the Lichtenstein procedure. It depicts two periods one until 2004 where the differences were non-significant and the second which starts with the study of Neumayer in 2004 until the present day where the Lichtenstein repair demonstrates a significantly lower recurrence rate. Interestingly, the high-quality RCT by Gutlic [29] did not influence the results significantly either way (Fig. 3).

## Discussion

This study shows that the TEP cohort had a higher recurrence rate and vascular injury rate than the Lichtenstein cohort. However, the TEP cohort had better outcomes in terms of haematoma formation rate, time to return to usual activities, and local paraesthesia compared with the Lichtenstein cohort. No difference was found between the 2 cohorts in terms of wound infection, persistent pain, and time to discharge.

The recurrence rate is difficult to explore, because it depends on varied follow-up periods [30]. The follow-up periods in this study also varied widely. Usually, the recurrence rate is estimated as twice the number of reoperations [31]. A Danish observational study reported that the reoperation rates following TEP laparoscopic hernioplasty and Lichtenstein hernioplasty were 3.3% and 2.4%, respectively [32]. It therefore suggests that the recurrence rates were around 6.6% and 4.8% for the laparoscopic and Lichtenstein hernioplasties, respectively. It is noteworthy that our study recorded recurrence rates of 6% and 4% for the TEP and Lichtenstein hernioplasties, respectively, which are similar to those of the above-mentioned study.

The reported incidence of clinically significant chronic persistent pain was 10–12% with a tendency to decrease with time [33, 34]. In this study, 11% of patients in the TEP cohort and 13% of the patients in the Lichtenstein cohort had chronic persistent pain, but no statistically significant differences were observed between the cohorts.

**Fig. 4:**
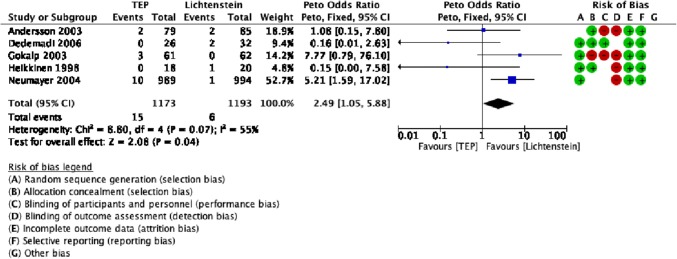
Forest plot of vascular injuries

The reported incidence of vascular injury during inguinal hernioplasty is 0.1–0.4% [35]. Data from the German registry Herniamed reported significantly more vascular injuries of 1.39% in TEP compared to 1.13% in TAPP [36]. In this study, the rate of vascular injuries was significantly higher in the TEP cohort at 1.3% compared to 0.5% in the Lichtenstein cohort (Fig. 4, Table 1).

Furthermore, the haematoma formation rate was significantly less in the TEP cohort than in the open cohort. However, the lack of a haematoma severity classification and a common haematoma definition that is clinically relevant for the laparoscopic and open approaches make the extrapolation of objective conclusions difficult. Other potential contributors to diagnostic bias are preperitoneal haematomas that may be of similar sizes to superficial haematomas of open procedures, but may not be as easily diagnosable as those of the open procedure [1].

Cumulative meta-analysis further supports the findings of traditional meta-analysis by demonstrating that from 2004 until present, the recurrence rate is significantly lower for Lichtenstein repair. Interestingly, the most recently published high-quality RCT by Gutlic [29] did not influence the accumulated evidence.

To the best of the authors’ knowledge, this is the most up-to-date study and the first cumulative meta-analysis with 21 included studies and 6573 enrolled patients compared to the previous meta-analysis with 14 studies and 3279 patients [3]. However, the results of this study should be interpreted with caution due to the study limitations. The overall quality of the included RCTs was poor; only 4 of the 21 studies blinded the participants and the personnel, and one of them blinded the outcome assessors (Table 2). The total sample was quite heterogeneous as it included patients with primary, recurrent, and bilateral hernias. In addition, the studies were conducted in single centres and the follow-up periods varied widely (Table 3). Therefore, national and institutional characteristics, underpowered and heterogeneous samples, performance, and detection bias may have influenced the results. However, the outcome measures described in this study provide contemporaneous comparative data to allow surgeons to discuss the potential risks, benefits, and alternative treatment options with patients considering their options for inguinal hernia repair.

**Table 3:** Study characteristics

Author (year)	Number of patients TEP-L	Age TEP-L	FU period (months)
Wright et al. (1995) [9]	60–60	63 ± 6.25 68 ± 6.5	NR
Heikkinen et al. (1998) [10]	18–20	51 ± 8.5 55.5 ± 10	NR
Gokalp et al. (2003) [11]	61–62	47 ± 10.75 45 ± 10.5	18
Andersson et al. (2003) [12] P&R	81–87	50 ± 9 49 ± 9	12
Colak et al. (2003) [13]	67–67	49.4 ± 14.25 51.6 ± 15.25	12
Lal et al. (2003) [14]	25–25	36.72 ± 12.08 37.8 ± 12.43	13
Bringman et al. (2003) [15]	92–103	55 ± 12 54 ± 11	20
Neumayer et al. (2004) [16]	989–994	58.6 ± 12.8 58.4 ± 12.7	24
Heikkinen et al. (2004) [17]	62–61	46 ± 12 48 ± 13	60
Lau et al. (2006) [18]	100–100	55 ± 15.5 56 ± 13.1 p = 0.583	12
Dedemadi et al. (2006) [19]	26–32	NR	36
Pokorny et al. (2007) [20]	36–69	48 ± 13.5 52 ± 16.25	36
Hallén et al. (2008) [21]	92–93	NR	87
Eklund et al. (2008) [23]	665–705	53 ± 9.6 52 ± 10.1	60
Kuhia et al. (2009) [24]	49–47	57.8 ± 12.6 55.8 ± 12	60
Hamza et al. (2010) [22]	25–25	34.91 ± 13 35.12 ± 10.1	6
Eker et al. (2012) [25]	336–324	55 56	60
Dhankhar et al. (2013) [26]	29–30	38.17 ± 11.53 43.20 ± 13.59	3
Wang et al. (2013) [27]	84–84	48.25 ± 17.09 52.12 ± 17.46	16
Moreno-Egea et al. (2014) [28]	106–102	NR	24
Gutlic et al. (2019) [29]	239–241	51 ± 12 54 ± 12	36
Pooled estimates	3242–3331 total 6573	OR = − 1.09 (− 2.33, 0.15), *p* = 0.09	

*TEP* total extraperitoneal, *L* Lichtenstein, *FU* follow-up

### Implications for research

To shed further light on the topic, multicentre RCTs with the following characteristics should be conducted: strict adherence to standards recommended in the Consolidated Standards of Reporting Trials (CONSORT) guidelines; comparison of primary, recurrent, and simultaneously performed bilateral hernias in separate patient groups; adequate sample power with predefined outcome measures critical for decision making according to the Grading of Recommendations Assessment, Development, and Evaluation system (GRADE); blind outcome assessors (although we acknowledge that this would be very challenging when assessing longer term outcomes); common methods of outcome assessment; and a follow-up period at least 3 years [37, 38].

## Electronic supplementary material

Below is the link to the electronic supplementary material.

Supplementary material 1 (DOCX 169 kb)

Supplementary material 2 (DOCX 280 kb)
