# Supplementary material for: Total extraperitoneal endoscopic hernioplasty (TEP) versus Lichtenstein hernioplasty: a systematic review by updated traditional and cumulative meta-analysis of randomised-controlled trials
Source: Hernia. 2019 Oct 10;23(6):1093–103. doi: 10.1007/s10029-019-02049-w (PMC6938473; doi:10.1007/s10029-019-02049-w)
Supplement: Supplementary file 1 — Supplementary material 1 (DOCX 169 kb) [file 10029_2019_2049_MOESM1_ESM.docx]

TEP vs Lichtenstein

CMA Recurences
