# Supplementary material for: Total extraperitoneal endoscopic hernioplasty (TEP) versus Lichtenstein hernioplasty: a systematic review by updated traditional and cumulative meta-analysis of randomised-controlled trials
Source: Hernia. 2019 Oct 10;23(6):1093–103. doi: 10.1007/s10029-019-02049-w (PMC6938473; doi:10.1007/s10029-019-02049-w)
Supplement: Supplementary file 2 — Supplementary material 2 (DOCX 280 kb) [file 10029_2019_2049_MOESM2_ESM.docx]

Persistent Pain

CMA
